# Unveiling Esophageal Candidiasis

**DOI:** 10.7759/cureus.79101

**Published:** 2025-02-16

**Authors:** Mariana R Laranjeira, Joana Q Gomes, Ana R Ferreira, Sofia Ramalheira

**Affiliations:** 1 Internal Medicine, Vila Nova de Gaia/Espinho Hospital Center, Vila Nova de Gaia, PRT; 2 Allergy and Immunology, Vila Nova de Gaia/Espinho Hospital Center, Vila Nova de Gaia, PRT; 3 Hematology and Oncology, Vila Nova de Gaia/Espinho Hospital Center, Vila Nova de Gaia, PRT

**Keywords:** esophageal candidiasis, esophagitis, human immunodeficiency virus infection, immunodeficiency, systemic mastocytosis

## Abstract

Esophageal candidiasis (EC) occurs more commonly in patients with human immunodeficiency virus (HIV) in the AIDS stage (CD4+ counts < 100 cells/ml). Other conditions of immunosuppression in which EC is more frequent include hematological malignancies, particularly receptors of hematopoietic precursors, solid cancers undergoing chemotherapy, and, less frequently, primary defects of the immune system. Although infrequent, EC can also occur in immunocompetent individuals. In these cases, the risk is higher in users of proton pump inhibitors, high-dose inhaled corticosteroids, and smokers.

The differential diagnosis includes other opportunistic infections (*Cytomegalovirus*, herpes simplex virus), drug esophagitis, gastroesophageal reflux disease, and/or eosinophilic esophagitis.

The clinical case presented aims, above all, to raise awareness of the multiplicity of etiologies of EC and not, as was the case in the past, to classify it as an AIDS-defining disease.

## Introduction

Esophageal candidiasis (EC) most commonly occurs in patients with human immunodeficiency virus (HIV) in the AIDS stage (CD4+ counts < 100 cells/ml). Other immunosuppressive conditions where EC is more frequent include hematologic malignancies, solid tumors, immunosuppressive treatments (notably chemotherapy), and, less frequently, primary immune deficiencies. Despite being uncommon, EC can also occur in immunocompetent individuals, where the risk is higher among users of proton pump inhibitors (PPIs), high-dose inhaled corticosteroids (ICS), and smokers. The rise in immune-related diseases has contributed to a higher incidence [1-4].

Species of *Candida* are part of the normal microbiota of the skin, oral cavity, nasal cavity, and the genitourinary and gastrointestinal tracts of healthy individuals, with *Candida albicans* constituting the esophageal commensal flora in 20% of individuals. Infection occurs when the innate and acquired immune mechanisms of the gastrointestinal tract responsible for inhibiting fungal proliferation are compromised, as happens in chemotherapy/radiotherapy for hematological disorders, primary or acquired immunosuppression, endocrinological disorders, or malnutrition [1,2]. Moreover, all conditions that lead to esophageal dysbiosis can alter local defense mechanisms and enhance infection, notably recurrent use of antibiotics, PPIs, and ICS [3,4].

Odynophagia is the most frequently reported symptom in EC; however, clinical presentation can vary widely from asymptomatic to nonspecific presentations with abdominal pain, dysphagia, and retrosternal discomfort. The concomitant presentation of oropharyngeal candidiasis and EC is common; however, the absence of oropharyngeal involvement does not exclude EC [3,4]. It is crucial to diagnose and treat this condition promptly to prevent complications such as esophageal ulceration, perforation, or chronic infection.

The differential diagnosis includes other opportunistic infections (such as *Cytomegalovirus* and herpes simplex virus), drug-induced esophagitis, gastroesophageal reflux disease, and/or eosinophilic esophagitis [1-4].

## Case presentation

**Figure 1:**
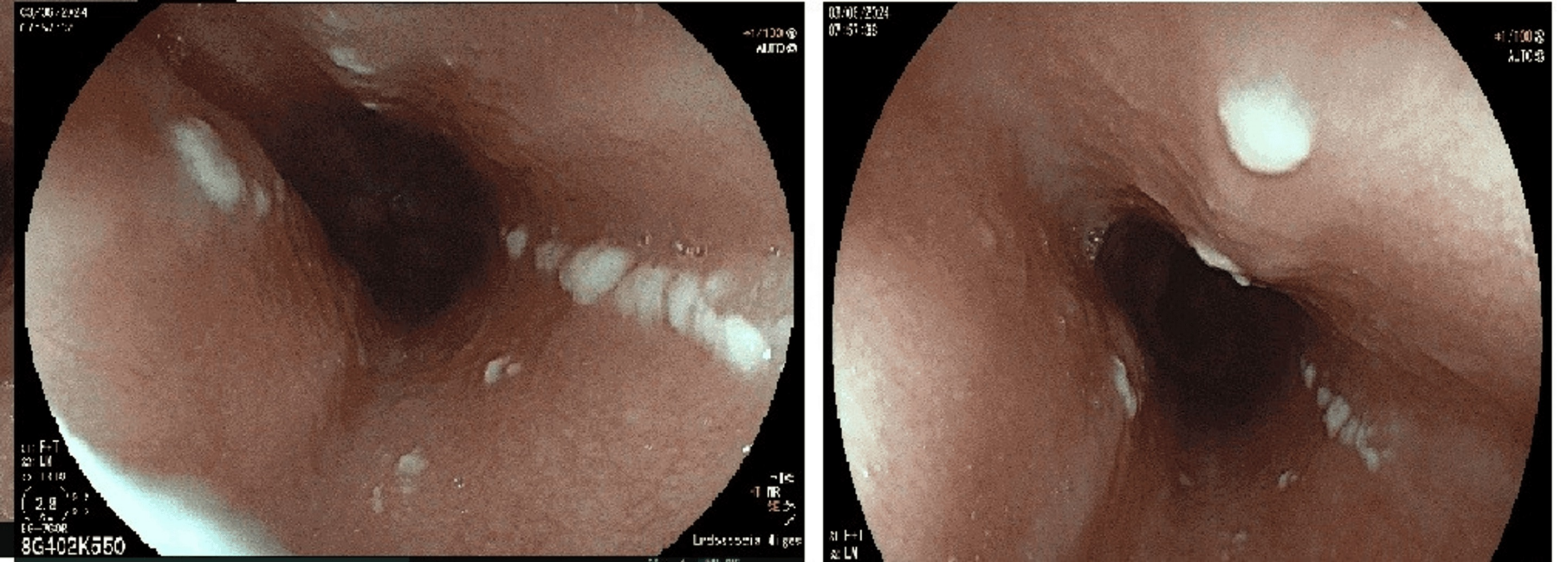
Upper gastrointestinal endoscopy. Whitish plaques were noted, suggestive of esophageal candidiasis, which were non-removable with a water jet.

We present a case of a 28-year-old woman with a medical history of indolent systemic mastocytosis monitored in the allergy and hemato-oncology outpatient clinic, central hypothyroidism monitored in the endocrinology outpatient clinic, and non-allergic rhinitis. She was regularly taking cetirizine, bilastine 20 mg, and levothyroxine 50 mcg. She sporadically used fluticasone furoate 27.2 mcg intranasally for periods. She visited her primary care physician due to retrosternal pain, anorexia, and a weight loss of 10% of her usual weight over three months. She denied recent infections, travel, high-risk sexual behavior, smoking, or intravenous drug use. She reported control of mast cell activation symptoms with her prescribed medication. Due to complaints of gastroesophageal reflux, she had empirically started PPIs in the previous four months. Due to significant weight loss, an upper gastrointestinal endoscopy (UGE) was requested, revealing imaging suggestive of esophageal candidiasis (Figure 1).

To clarify the occurrence of esophageal candidiasis in an apparently immunocompetent patient, a range of complementary diagnostic tests were requested to identify possible risk conditions that could justify it. Noteworthy diagnostic steps included viral markers and serologies. The patient was HIV negative and had a serological profile consistent with past *Cytomegalovirus* (CMV) and Epstein-Barr virus (EBV) infection, immunity from hepatitis B virus (HBV) vaccination, negative hepatitis C virus (HCV) antibodies, and serological profile indicating no exposure to herpes simplex virus (HSV)-1 or HSV-2. These results are presented in Table 1.

**Table 1:** Viral serologies. The presented results confirm that the patient had no exposure to HSV or HIV. The serological profile is compatible with past infection with EBV and CMV.

Table 1	Patient’s values	Normal values
Anti-hepatitis C virus (HCV) antibody	0.1	Negative: <0.90; positive: >1.10
Anti-HIV antibodies	0.1	Negative: <0.90; positive: >1.10
Hepatitis B surface antigen	0.5	Negative: <0.90; positive: >1.10
Anti-herpes simplex virus (HSV)-1 IgM	<0.11	Negative: <0.90; positive: >1.10
Anti-HSV-1 IgG	<0.19	Negative: <0.90; positive: >1.10
Anti-HSV-2 IgG	<0.10	Negative: <0.90; positive: >1.10
Anti-HSV-2 IgM	<0.10	Negative: <0.90; positive: >1.10
Anti-Cytomegalovirus (CMV) IgG	57.3 UA/ml	Reactive: >6.0; non-reactive: <6.0
Anti-CMV IgM	0.12	Reactive: >1.00; non-reactive: <0.85
Anti-Epstein-Barr virus (EBV) IgG	61.06 U/ml	Reactive: >1.00; non-reactive: <0.75
Anti-EBV IgM	0.02 U/ml	Reactive: >1.00; non-reactive: <0.50

**Figure 2:**
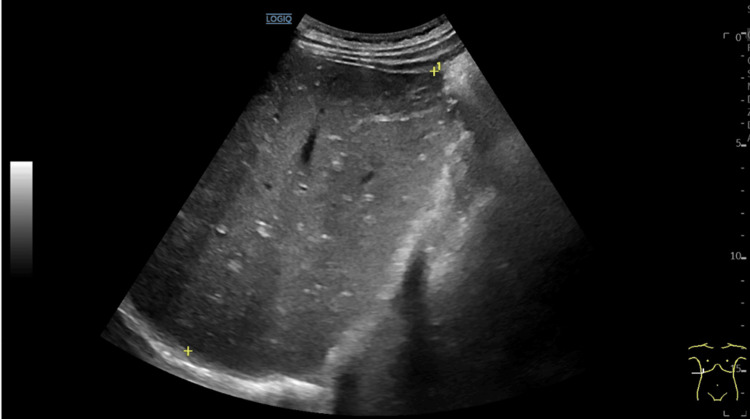
Abdominal ultrasound (A). Liver with elongated morphology (16.5 cm), regular contours, and homogeneous parenchyma. Thin-walled gallbladder, without lithiasis. No dilatation of the bile ducts. The yellow asterisks represent the longitudinal length of the liver.

**Figure 3:**
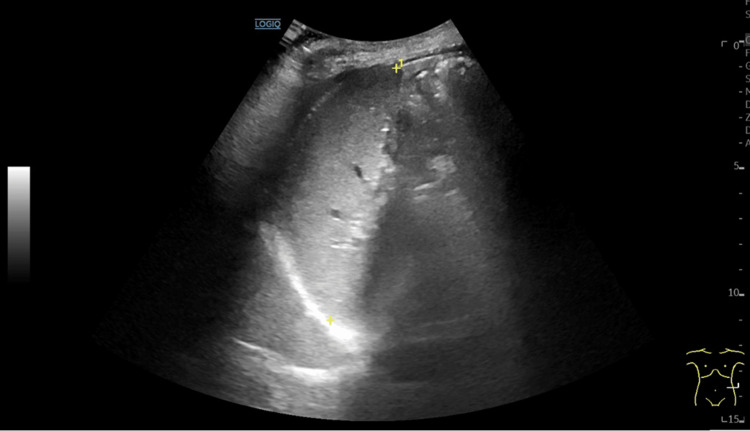
Abdominal ultrasound (B). The spleen had normal dimensions (10.3 cm), regular contours, and homogeneous parenchyma. The yellow asterisks represent the longitudinal length of the spleen.

**Figure 4:**
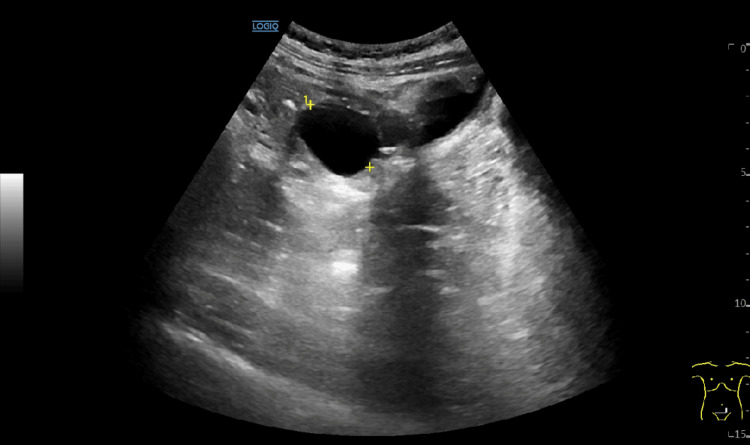
Gynecological ultrasound. Gynecological ultrasound revealed two adnexal cysts on the left measuring 2.7 and 3.7 cm, of simple appearance, probably functional.

Abdominal, gynecological, thyroid, and breast ultrasounds showed no signs of neoplasia or hepatosplenomegaly (Figures 2-4).

A study of lymphocyte subpopulations was also performed showing no lymphopenia. No complement deficiency or hypogammaglobulinemia was noted, as seen in Tables 2, 3.

**Table 2:** Differential leukocyte and lymphocyte count. Changes in differential lymphocyte count were not considered significant, so they would not justify dysbiosis of the esophageal microbiome. ↑ - values above the reference values. ↓ - values below the reference values.

White blood cells	Patient’s values	Normal values (x10^9^/L)
Leukocytes	6.80	4.0-10.0
Neutrophils	3.09	1.5-8.0
CD3+ T lymphocytes	85.8%, 1879 cells/ul (↑)	1100-1700 cells/ul
CD3+/CD4+ T lymphocytes	58.1%, 1272 cells/ul (↑)	700-1100 cells/ul
CD3+/CD8+ T lymphocytes	23.6%, 517 cells/ul	500-900 cells/ul
CD4+/CD8+ ratio	2.46	1.1-4.0
CD19+ B lymphocytes	7.5%, 164 cells/ul (↓)	200-400 cells/ul
CD3-/CD56+ natural killers lymphocytes	6.6%, 145 cells/ul (↓)	200-400 cells/ul
Eosinophils	0.11	0.0-0.30
Monocytes	0.46	0.0-1.20
Basophils	0.02	0.0-0.30

**Table 3:** Serum immunoglobulins assay.

Serum immunoglobulins	Patient’s values	Normal values
Immunoglobulin M	119 mg/dl	40-248 mg/dl
Immunoglobulin G	890 mg/dl	680-1450 mg/dl
Immunoglobulin A	251 mg/dl	61-374 mg/dl

Serum tryptase was at 10x of the normal range, without acute mastocytosis exacerbations, consistent with usual levels. No deficiencies in vitamin B12, folic acid, or iron were noted. In Table 4, we present the patient's lab values.

**Table 4:** Vitamin, iron, and tryptase assays.

	Patient’s values	Normal values
Folic acid	5.2 ng/ml	4.6-18.7 ng/ml
Vitamin B12	312.0 pg/ml	197-771 pg/ml
Serum iron	108 ug/dl	37-145 ug/dl
Total binding iron capacity	426 ug/dl	228-428 ug/dl
Ferritin	15.7 ng/ml	13-150 ng/ml
Transferrin saturation	25.4%	15-55%
Serum tryptase	97.5 ug/L	1-15.0 ug/L

The pathological study confirmed the following diagnosis: “distal esophagus with yeast-like structures and spores, morphologically compatible with *Candida*, confirmed after periodic acid-Schiff (PAS) staining. *H. pylori* testing negative. No signs of gastritis, peptic ulcer, or inflammatory infiltrate.”

After excluding other causes and given the dysbiosis induced by PPI, this was assumed to be the etiology, and the patient completed a cycle of fluconazole 400 mg daily for three weeks, discontinuing the PPI. After three months, a follow-up endoscopic study was performed, revealing no histological findings compatible with persistent infection. The patient also reported symptomatic improvement, with a weight gain of about 2 kg, reduced retrosternal pain, and increased appetite.

## Discussion

EC is still considered one of the most challenging and common infections of the esophagus. It is important to note that the infection tends to be less severe and more easily treatable compared to cases in immunocompromised individuals [5,6].

A detailed understanding of the disease is essential for effective therapeutic management, as the prognosis can vary depending on the causative agent and the patient's overall health. In some cases, the infection can be treated with oral antifungals, while in others, more intensive approaches, including intravenous antifungals, may be necessary [5,6].

On the other hand, candidiasis may only represent a superficial colonization of the esophagus, especially in individuals with no significant symptoms or those with a resolving underlying condition. In such cases, aggressive treatment may no longer be necessary once the root cause (e.g., HIV, diabetes, and immunosuppressive therapy) is under control. The focus should shift to managing the underlying condition and monitoring the individual for symptoms. If the patient is asymptomatic and the infection does not cause complications, the approach may transition to observation, with treatment reserved for instances of active infection or clinical symptoms. In patients in whom candidiasis represents only a superficial colonization, without symptoms and not an impactful disease, the possibility of not instituting treatment with antifungals should always be considered due to a greater risk of drug resistance, reserving its use for cases in which the patient's general condition and quality of life deteriorates [7,8].

This clinical case motivated further investigation of cases of EC later diagnosed, with more cases being found not in HIV patients but those with advanced-stage neoplasms or undergoing chemotherapy/radiotherapy. It should be noted, however, that these data may be biased given the greater interest in the area and the active and incessant search for diagnosis.

## Conclusions

In summary, early diagnosis and proper treatment of EC are vital, particularly in immunocompromised individuals. Timely intervention can significantly improve the patient's quality of life and reduce the risk of further systemic spread. The association with HIV/AIDS should not lead to stigma, and the disease should be understood as a result of immune system dysfunction. Managing symptoms and addressing the underlying cause is essential to improving patient outcomes and quality of life.

This clinical case aimed to raise attention to the nonspecific symptoms of EC, from the absence of symptoms to abdominal pain, dysphagia, or retrosternal pain, to recognize other risk factors for the development of EC, namely, immunosuppression, and to reduce the stigma associated with the disease.
